# Origin of the *oxa235* carbapenem resistance gene found in transposon Tn*6252*

**DOI:** 10.1093/jac/dkac013

**Published:** 2022-01-30

**Authors:** Stephanie J. Ambrose, Benjamin A. Evans, Ruth M. Hall

**Affiliations:** 1 School of Life and Environmental Sciences, The University of Sydney, Sydney, NSW 2006, Australia; 2 Norwich Medical School, University of East Anglia, Norwich, UK

An increasing number of antibiotic resistance genes found in the mobile gene pool of *Acinetobacter* species are part of transposons that are mobilized by the insertion sequence ISAba1. ISAba1 includes a strong, outward-facing promoter, originally identified by Segal *et al*.^1^ and later re-positioned,^2^ and overexpression from this promoter can convert intrinsic genes into resistance genes.^3,4^ For example, the widespread *oxa23* carbapenem resistance gene is known to originate from an intrinsic gene encoding a class D β-lactamase that is found in the chromosome of *Acinetobacter radioresistens.*^4^ For clarity and simplicity, the *A. radioresistens* gene is referred to using the designation *oxaAr*,^5^ a term that encompasses all chromosomal alleles. An *oxaAr* variant has been mobilized twice from the *A. radioresistens* chromosome by an ISAba1 located upstream to create Tn*2008*A and Tn*2008*B (see Nigro and Hall^5^). Subsequently, larger compound transposons bounded by two copies of ISAba1 in inverse (Tn*2006*) or direct (Tn*2009*) orientation have arisen from Tn*2008*B.^5^ Though *oxaAr* is not known to confer resistance to carbapenem antibiotics, *oxa23* in its new context is expressed from the strong outward-facing ISAba1 promoter and confers resistance. ISAba1 has also mobilized the intrinsic *ampC* gene from an *Acinetobacter baumannii* to form Tn*6168*, which confers resistance to third-generation cephalosporins due to increased expression driven by the promoter in ISAba1.^6^ Tn*6168* has been found, in addition to the intrinsic *ampC* gene, in the chromosome of a group of *A. baumannii* ST1^IP^ isolates^7^ and in a plasmid in an *A. baumannii* ST49^IP^ outbreak.^8^

Tn*6252*, which includes the *oxa235* gene bounded by inversely oriented copies of ISAba1, was first reported in the chromosome of ST10^IP^ isolate LAC-4.^9^ The upstream ISAba1 is oriented such that the strong promoter internal to ISAba1 drives expression of the *oxa235* gene (Figure 1a). The cloned *oxa235* gene (originally called *bla*_OXA-235_) had been shown to confer modest levels of resistance to carbapenem antibiotics.^10^ Later, Tn*6252* was also found in the potentially conjugative plasmid pRCH51-3 (GenBank accession number KY216144) and was responsible for the reduced susceptibility to carbapenems of a sporadic *A. baumannii* isolate RCH51.^11^ Tn*6252* was also found in a GC2 outbreak.^12^ In each case, the 3267 bp Tn*6252* is surrounded by a 9 bp target site duplication (TSD), as is typical of ISAba1 transposition, indicating that it is an active transposon.

**Figure 1.**
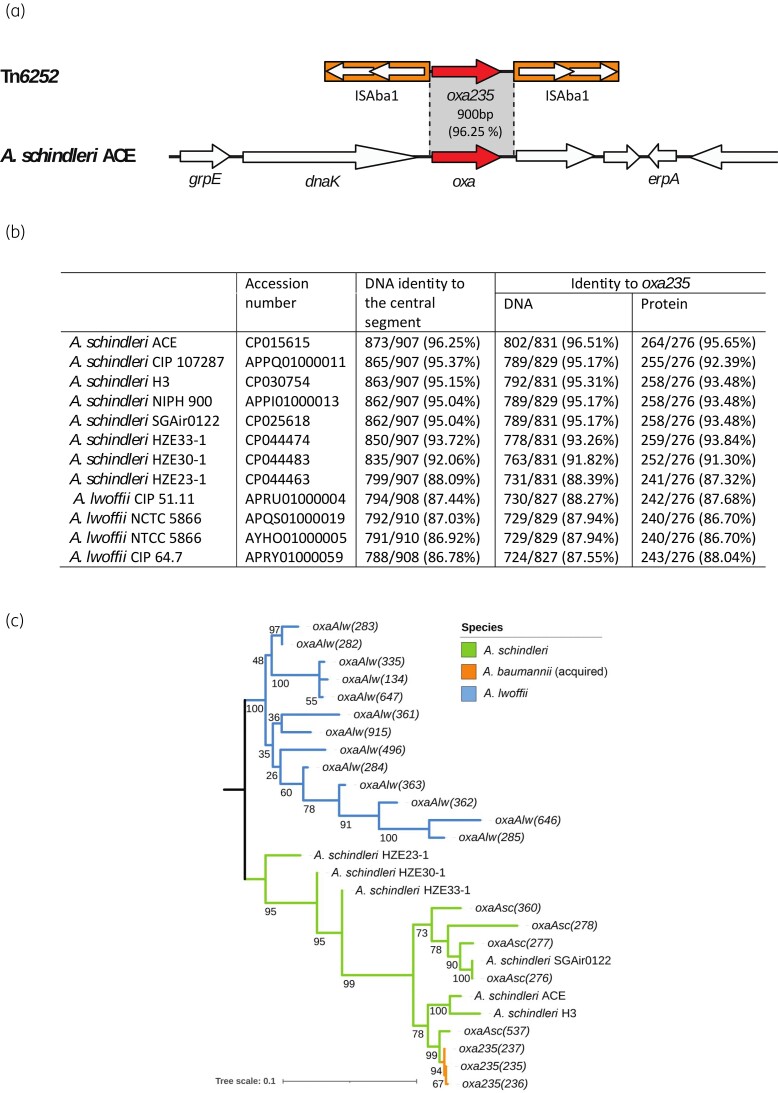
Origin of *oxa235*. (a) Comparison of Tn*6252* with *A. schindleri* ACE chromosomal sequence. The extent and orientation of genes are indicated by arrows with the gene names below. The chromosomal *oxa* gene and *oxa235* are shown in red, while other genes and open reading frames are shown in white. ISAba1 is shown as an orange box with arrows inside to indicate the transposase genes. Grey shading indicates regions shared between the two sequences. Drawn to scale from GenBank accession numbers CP015615 (*A. schindleri* ACE) and KY216144 (Tn*6252*). (b) Comparison of *A. schindleri* and *A. lwoffi* chromosomal sequences with *oxa235* and the central segment of Tn*6252*. (c) Maximum likelihood tree of *oxa* genes from the *oxaAlw*, *oxaAsc* and *oxa235* groups. In Geneious Prime, nucleotide sequences were aligned using Clustal Omega with default settings and the tree was constructed using PhyML with the GTR substitution model optimized for topology/length/rate, and confidence was assessed by performing 100 bootstraps. Percentage support from bootstrapping is shown on the branches. Where *oxa* allele numbers have been assigned by the NCBI Bacterial Antimicrobial Resistance Reference Gene Database, these are given in parentheses. Where no allele number has been assigned, the name of the strain that the gene derived from has been given. This figure appears in colour in the online version of *JAC* and in black and white in the print version of *JAC*.

Here, we have examined the distribution of Tn*6252* and the origin of the *oxa235* gene. Though Tn*6252* is not often encountered (21 entries were found in the GenBank nucleotide database as of August 2021), examination of the locations of Tn*6252* in those sequences revealed 12 positions in the chromosome and 5 in plasmids, usually flanked by a 9 bp duplication indicative of movement.

The similarity (85% identity) of OXA-235 [and two minor variants (OXA-236 and OXA-237) with a single amino acid difference] to OXA-134, which was encoded by an intrinsic gene in the chromosome of an *Acinetobacter lwoffii* isolate, suggesting a possible chromosomal source, had been noted.^10^ Here, the closest match of 98.92% identity was to an intrinsic gene encoding a class D β-lactamase (KX360744) reported to be from the chromosome of an *Acinetobacter schindleri* isolate. The segment of DNA that includes the *oxa235* gene found between the ISAba1 copies was found to share 92%–96% identity to the corresponding region in most of the *A. schindleri* chromosomes for which complete or draft sequences are available and was more distantly related to the corresponding region in *A. lwoffii* genomes (Figure 1b).

For simplicity, the *A. schindleri* gene, covering all alleles, is referred to here as *oxaAsc.* A phylogeny of the OXA variants encoded by the *oxa235* gene (OXA-235, OXA-236 and OXA-237) and those currently assigned to *A. lwoffii* or *A. schindleri* in RefSeq (Figure 1c) also revealed a clear separation of the alleles currently designated as ‘OXA-134 family’ into two groups corresponding to those encoded by *A. lwoffii* and *A. schindleri* chromosomes and the *oxa235* alleles clearly group with those derived from *A. schindleri*. The species assignment of the *A. schindleri* genomes was confirmed using ribosomal RNA gene sequences (Figure S1, available as Supplementary data at *JAC* Online). The first recorded allele of the *oxaAsc* gene in RefSeq (https://www.ncbi.nlm.nih.gov/refseq/) is designated *bla*_OXA-276_ and we recommend that the two groups currently designated ‘OXA-134 family’, but corresponding to different species origins, should be separated into OXA-134 and OXA-276 groups.

We also recommend that context should be considered in order to distinguish the intrinsic chromosomally located genes that are not known to confer resistance to carbapenems from the mobilized *oxa23* and *oxa235* genes that have spread into other species and now because of their context confer resistance to carbapenem antibiotics.

Recently, we reported mobilization of the chromosomal *folA* gene of an *A. schindleri* isolate by ISAba60 to generate the *dfrA44* trimethoprim resistance gene.^13^ However, the *dfrA44* gene and surrounds match a region in the chromosomes of the completely sequenced *A. schindleri* isolates with >98.3% identity. Hence, closer matches for the *oxa235* gene may be found in the future.

## Supplementary Material

dkac013_Supplementary_Data

## Supplementary data

Figure S1 is available as Supplementary data at *JAC* Online.
