# Supplementary material for: Origin of the oxa235 carbapenem resistance gene found in transposon Tn6252
Source: J Antimicrob Chemother. 2022 Jan 30;77(4):1197–9. doi: 10.1093/jac/dkac013 (PMC9165733; doi:10.1093/jac/dkac013)
Supplement: dkac013_Supplementary_Data [file dkac013_supplementary_data.docx]

**Supplementary data**


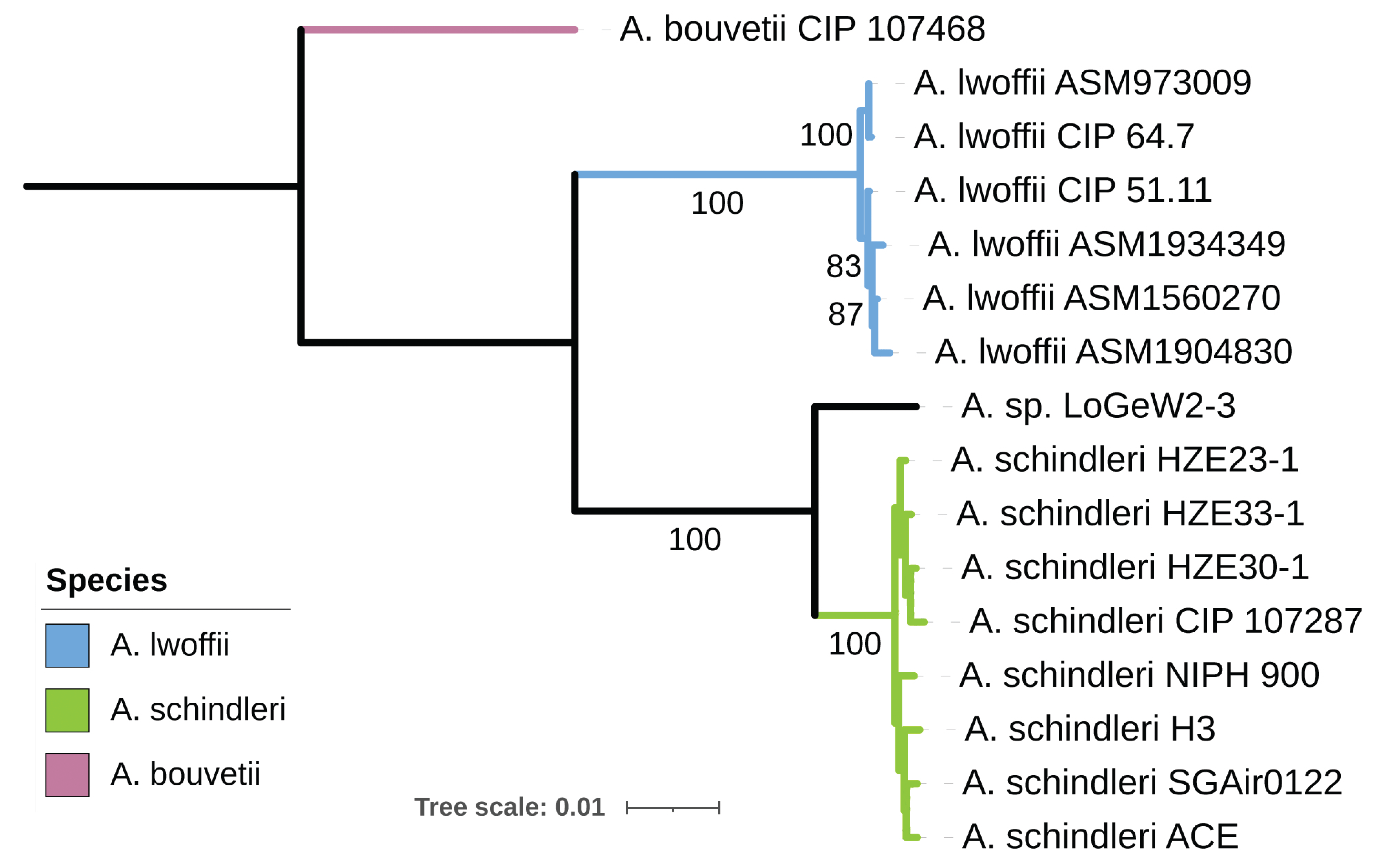


**Figure S1.** Maximum likelihood tree of *A. schindleri* and *A. lwoffii* strains. In the programme Geneious Prime, the nucleotide sequences of 15 ribosomal RNA genes (L2, L3, L4, L5, L6, L14, L16, L18, L22, L24, S3, S8, S10, S17, S19) for each strain were concatenated and aligned using Clustal Omega with default settings, and the tree was estimated using PhyML with the GTR substitution model, optimize topology/length/rate, and confidence was assessed by performing 100 bootstraps. Percentage support from bootstrapping is shown on the branches. Only bootstrapping values greater than 80% are shown for clarity. *Acinetobacter bouvetii* CIP 107468 was included as an outgroup and used to root the tree. Strain *Acinetobacter* species LoGeW2-3 was included as it is known to cluster near, but distinct from, *A. schindleri* strains (Evans, unpublished data).
